# Complement and coagulation cascade cross-talk in endometriosis and the potential of Janus Kinase inhibitors—a network meta-analysis

**DOI:** 10.3389/fimmu.2025.1619434

**Published:** 2025-07-08

**Authors:** Monika Golinska, Aleksander Rycerz, Matylda Sobczak, Jedrzej Chrzanowski, Konrad Stawiski, Wojciech Fendler

**Affiliations:** ^1^ Department of Biostatistics and Translational Medicine, Medical University of Lodz, Lodz, Poland; ^2^ Cancer Research UK Cambridge Institute (CRUK CI), University of Cambridge, Cambridge, United Kingdom; ^3^ Department of Radiation Oncology, Dana-Farber Cancer Institute, Boston, MA, United States; ^4^ Medical Research Agency, Warsaw, Poland

**Keywords:** endometriosis, eutopic and ectopic endometrium, network meta-analysis, complement and coagulation, mast cells, Janus kinase (JAK) inhibitors

## Abstract

**Background:**

Molecular events that drive endometriosis (EM) and cause accompanying immune deregulation remain elusive. Our purpose was to identify key pathways involved in lesion formation across diverse populations and to detect transcriptomic changes in eutopic endometrium that accompany EM.

**Methods:**

We searched Gene Expression Omnibus and ArrayExpress and performed differential gene expression analysis and a network meta-analysis on nine qualifying datasets. Those contained transcriptomic data on 114 ectopic endometrium samples (EL), 138 eutopic endometrium samples from women with endometriosis (EEM), and 79 eutopic endometrium samples from women without endometriosis (EH). Gene ontology and enrichment analysis were performed in DAVID, Metascape, and Cytoscape, and drug repurposing was done in CMap.

**Results:**

EEM compared to EH upregulated *CCL21* and downregulated *BIRC3*, *CEL*, and *LEFTY1* genes (|log_2_FC| > 0.5, *p* < 0.05). EL showed increased expression of complement and serpin genes (EL vs. EEM: *C7*, logFC = 3.38, *p* < 0.0001; *C3*, logFC = 2.40, *p* < 0.0001; *SERPINE1*, logFC = 1.02, *p* < 0.05; *SERPINE2*, logFC = 1.54, *p* < 0.001) and mast cell markers (EL vs. EEM: *CPA3*, logFC = 1.54, *p* < 0.0001; *KIT*, logFC = 0.74, *p* < 0.001). Functional enrichment analysis highlighted complement and coagulation, inflammation, angiogenesis, and extracellular matrix remodeling as drivers of endometriosis. Pharmacogenomic analysis indicated Janus kinase (JAK), cyclin-dependent kinase (CDK), and topoisomerase inhibitors as therapy targets.

**Conclusion:**

Our results suggest an interplay between complement and coagulation, mast cells, extracellular matrix remodeling, and the JAK/STAT3 pathway in endometriosis. We underscore the significance of complement C3 and propose JAK inhibitors as therapy candidates. Detected expression differences between EEM and EH are important for the development of diagnosis via endometrial biopsy.

## 1 Introduction

Immune system deregulation is a well-accepted phenomenon in endometriosis, and various inflammatory phenotypes have been associated with increased risk of this condition (1–3). To date, pathways contributing to the immune imbalance remain elusive. Lack of knowledge on the key processes that drive endometriosis hinders its early detection and therapy development. There is a need to define those molecular events and to understand how they interact to foster peritoneal inflammation.

Endometriosis is a chronic and complex disease currently showing a median diagnostic delay of 7–9 years (4–7). There has been significant progress in the development of endometriosis imaging protocols (8); however, laparoscopy remains a gold standard for final diagnosis. There is a need to explore the less invasive endometrial biopsy option. To consider this strategy, in-depth knowledge of the molecular differences in the eutopic endometrium of healthy controls and women with endometriosis is needed.

There has been significant progress in the development of endometriosis imaging protocols (8); however, laparoscopy remains a gold standard for final diagnosis. There is a need to explore the less invasive endometrial biopsy option. To consider this strategy, in-depth knowledge of the molecular differences in the eutopic endometrium of healthy controls and women with endometriosis needs to be obtained. Recent evidence shows the potential of stromal cells from menstrual discharge of women with endometriosis to initiate lesion growth in mice, thus highlighting the role of endometrial seeding (9). It was shown that eutopic endometria of women with and without endometriosis displayed different profiles of infiltrating immune cells. Ectopic endometrium tissue from women with endometriosis showed increased amounts of CD8+ T cells and CD56+ NK cells but decreased numbers of CD163+ macrophages, and those findings were correlated with increased risk of infertility (10). A detailed understanding of the alterations in eutopic endometrium that occur during endometriosis development will further shed light on the process of endometrial seeding and contribute to the development of endometrial biopsy as a diagnostic tool.

Several attempts have been made at delineating disease biomarkers, but to date this has not yet proven successful. Various omics technologies enabled identification of key genes related to the pathophysiology of endometriosis. However, a consensus has not yet been reached, and we are still missing the focal points on which to concentrate the therapeutic endeavors. A multi-cohort analysis is needed to address the issue in an unbiased and comprehensive manner.

In this article, we aimed to better understand complex events that underlie endometriotic lesion formation and progression. To achieve this, we systematically reviewed endometriosis data and performed network meta-analysis on chosen datasets. We generated a transcriptomic profile of endometriosis, determined the key pathways involved in lesion formation, and explored possible drug candidates for endometriosis therapy.

## 2 Materials and methods

### 2.1 Search strategy and study selection

The dataset search was conducted in public repositories Gene Expression Omnibus (GEO) and ArrayExpress to ensure that no relevant studies were missed. A systematic approach was employed to assess the risk of bias throughout both the dataset selection process and subsequent analyses.

Datasets were retrieved from the GEO using search terms “endometriosis” and “Homo sapiens” and filtered with terms “expression profiling by array” or “expression profiling by high-throughput sequencing.” MEDLINE and PubMed were searched for publications that correspond to those publicly deposited datasets.

The studies were identified using the following PICOS principle: Patients = patients with or without endometriosis, Intervention = bulk RNA performed on excised tissue, Comparison = dividing patients into those with endometriosis or without based on laparoscopic findings, Outcome = differential gene expression, and Study design = transcriptomic bulk RNA studies. Studies included in the analysis had to contain at least two tissues of interest: ectopic endometrium—endometrial lesion (EL), eutopic endometrium from women without endometriosis (EH), or eutopic endometrium from women with endometriosis (EEM).

**Figure 1:**
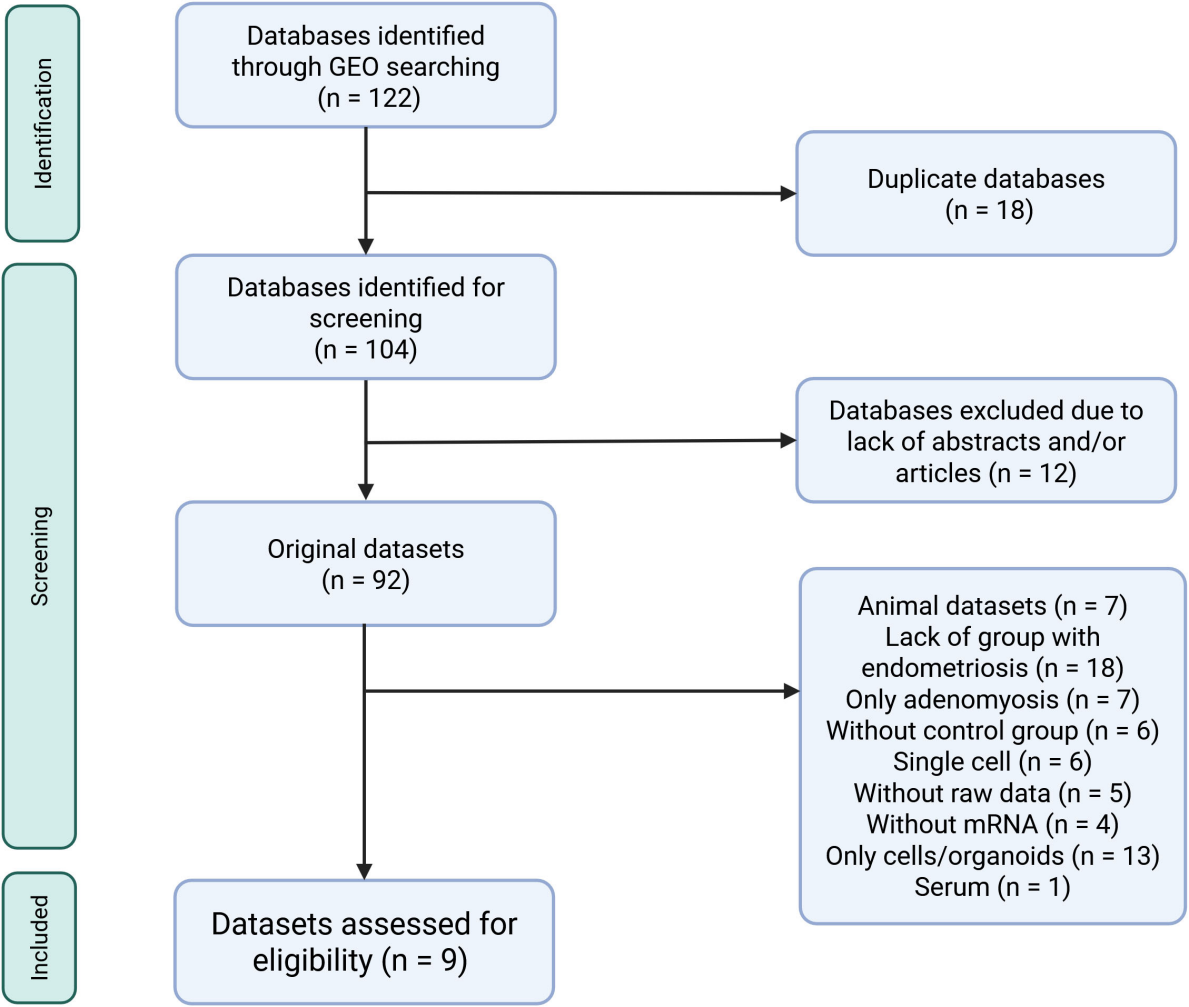
Flow diagram showing the selection process of databases retrieved from the Gene Expression Omnibus search for transcriptomic data comparing eutopic and ectopic endometrial tissue. All datasets from ArrayExpress were also deposited in Gene Expression Omnibus thus they were not further considered in the selection process. Created in https://BioRender.com.

Inclusion criteria were predefined and stringent to minimize the risk of bias, focusing on datasets using RNA sequencing (RNA-seq) or microarray technologies with available raw data. Transcriptomic analysis had to be performed directly on human endometrial tissue that had not been subjected to any manipulation or cell isolation prior to RNA extraction. Samples had to be taken from patients not on hormonal treatment in the 3 months preceding tissue collection. The presence or absence of endometriosis had to be confirmed with laparoscopy for samples to be included in our study. Only datasets with accompanying publications were considered to ensure all information about samples was available. Datasets with incomplete information were excluded to reduce variability and minimize errors. The database selection flowchart and a full list of inclusion/exclusion criteria are summarized in **Figure 1** and **Table 1**. Two independent reviewers screened datasets for relevance, and any discrepancies were resolved in discussion with a third reviewer. The study protocol was registered in PROSPERO (ID CRD42024548098).

**Table 1:** Inclusion and exclusion criteria for selection of GEO datasets.

Inclusion criteria
Transcriptomic datasets containing endometriosis and a control group
Accompanied by the corresponding publication
The presence or absence of endometriosis confirmed via laparoscopic procedure
Patients not undergoing hormonal treatment three months prior to tissue collection
Transcriptomic analysis performed directly on endometrial human tissue
Exclusion criteria
Tissue processing post excision (for further cell isolation or single cell analysis)
Adenomyosis included as endometriosis group
ScRNA-seq analysis
Lack of whole genome mRNA sequencing and or profiling
Sequencing or profiling performed on cells or organoids isolated from tissue
Lack of raw data deposited in GEO

### 2.2 Data extraction and differential gene expression

Each dataset was analyzed individually to ensure that data-specific preprocessing and normalization steps were applied appropriately. For microarray datasets, the raw data files were retrieved from the GEO repository using the R package “GEOquery” (11). The preprocessing of microarray data was conducted following the manufacturer’s protocols. Background correction and quantile normalization were applied for all array data. To adjust for differences in library size and transcript length, the raw read counts from RNA-seq datasets were normalized and scaled using the average transcript length for each sample. Following this, library size normalization was performed using the trimmed mean of M-values (TMM). After preprocessing, group comparisons were conducted on the normalized datasets to identify differentially expressed genes (DEGs) between experimental groups. For this purpose, the “limma” package in R was utilized. In the meta-analysis, we used logFC values and standard errors estimated with the limma package to calculate combined effect sizes. The estimation of standard errors incorporates empirical Bayes moderation, which provides more stable variance estimates compared to standard t-tests. The analysis was exploratory in nature, with the primary goal of identifying potentially differentially expressed genes that could be candidates for further investigation. Therefore, at this stage, no correction for multiple testing was applied. The results from this step served as a basis for subsequent functional analyses (pathway and pharmacogenomics analysis), in which appropriate methods accounting for multiple testing correction were employed. Additionally, the expression of significantly differentially expressed genes (defined as logFC > |0.5| and *p*-value < 0.05) showed consistent patterns across the individual datasets.

### 2.3 Network meta-analysis

Network meta-analysis on gene expression was performed using the “netmeta” package (12). This approach allows for the integration of information from multiple comparisons, even when direct comparisons between all datasets are not available, providing a comprehensive and robust analysis of the data. In the context of this study, network meta-analysis was used to compare gene expression levels across datasets systematically. Although meta-analysis allows for the determination of both direct and indirect effects, in our subsequent analyses, we focused on the combined effect to maximize the quality of the analyzed data and reduce the influence of less reliable direct or indirect effects. For investigated difference measurement, we used logFC and its corresponding standard error. These were interpreted as the mean difference and the standard error of the mean difference, respectively, which are widely used metrics in comparative gene expression studies. This standardization ensures that the results are both interpretable and comparable across datasets.

We performed 7,664 network meta-analysis for genes that occurred in each of the datasets included in the study. Genes with a *p*-value < 0.05 were considered statistically significant, and a |logFC| > 0.5 was used to filter genes with biologically meaningful changes in expression. This relatively low threshold was chosen to avoid missing potentially important differences. Choosing |logFC| > 0.5 may potentially result in the inclusion of genes that were influenced by random or systematic errors and biopsy quality if only one study was taken into consideration. Here, the biological significance of obtained differentially expressed genes is validated by the comparison across nine different datasets.

### 2.4 Risk of bias

To reduce the risk of bias, we included studies with raw data deposited and results published in peer-reviewed journals. Information from the accompanying publication was used to ascertain the quality of the study and to identify if the absence of endometriosis was properly determined and to confirm that tissue did not undergo any manipulation prior to RNA isolation. Heterogeneity was evaluated using *I*² statistics and Cochran’s Q-test, while sensitivity analyses validated the robustness of findings. Funnel plots were generated to assess bias. These measures ensured a thorough evaluation of potential biases, enhancing the reliability and validity of the meta-analytic findings. *I*² statistics and Cochran’s Q-test values are included in the **Supplementary Dataset**.

### 2.5 Gene ontology and pathway analysis

The list of DEGs obtained from the network meta-analysis was submitted to DAVID (13) for gene ontology and KEGG and Reactome pathways analysis. For functional clustering, we applied a cutoff enrichment score of >2.5, *p* < 0.05, and medium classification stringency. The same list of DEGs was analyzed in Metascape (14) v3.5.2024.0101, and the most enriched terms were visualized in Cytoscape (15) v3.10.2.

### 2.6 Computational pharmacogenomics

To identify pharmacological compounds likely to reverse the endometriosis gene signature, we queried the drug repurposing reference database, CMap (16). We submitted a list of 150 upregulated and 150 downregulated genes that had the highest combined logFC for EL versus EH and EL versus EEM comparisons and *p* < 0.05. The CMap analysis reports a median tau score, which represents connectivity strength between the submitted list of DEGs and thousands of compounds and perturbagens tested on human cell lines. A median tau score of above 90 or below −90 is considered to represent a high connectivity.

## 3 Results

### 3.1 Characteristics of chosen studies

Nine datasets met the inclusion criteria (**Table 1**; **Figure 1**) and were included in the analysis (**Table 2**). Those contained transcriptomic data on 114 ectopic endometrium samples (EL), 138 eutopic endometrium samples from women with endometriosis (EEM), and 79 eutopic endometrium samples from women without endometriosis (EH). The absence of endometriosis in the healthy (EH) group had to be confirmed during the laparoscopic procedure. EEM and EL samples were either obtained from the same person (in studies: GSE25628, GSE37837, and GSE7305) or from different individuals (in study GSE141549). Tissues were collected on three different continents and encompassed all types and stages of endometriosis (clinical data in **Table 3**).

**Table 2:** Characteristics of GEO datasets chosen for the network meta-analysis.

GEO accession number	Method	Number of detected genes	Ectopic endometrium (EL, n = 114)	Eutopic endometrium from patients with endometriosis (EEM, n = 138)	Eutopic endometrium from patients without endometriosis/ healthy control (EH, n = 79)
GSE232713 (90)	High- throughput sequencing	17488	–	7	7
GSE153740 & GSE153739 (91)	High- throughput sequencing	16840 & 17449	–	4 & 4	4 & 3
GSE141549 (92)	Microarrays	19746	79	49	21
GSE134056 (93)	High- throughput sequencing	18885	–	16	22
GSE25628 (94)	Microarrays	12644	7	9	6
GSE37837 (95)	Microarrays	21094	18	18	–
GSE6364 (96)	Microarrays	20857	–	21	16
GSE7305 (97)	Microarrays	20857	10	10	–

**Table 3:** Clinical characteristics of patients per dataset.

GEO accession number	Author	Age: mean (range)	Type of endometriosis	Stage of endometriosis	Cycle phase	Fertility	Ethnicity	Collection site
GSE232713	Hiao-Yan Li	33.93 (28–38)	Not provided	Not provided	Secretory (14)	Infertile (14)	Not provided	China
GSE153739 & GSE153740 ***	Kashmira Bane	31.70 (Not provided)	OE (4) PE (3) OE, PE (1)	II* III* IV*	Secretory (8) Proliferative (7)	Fertile* Infertile*	Not provided	India
GSE141549	Michael Gabriel	33.43 (21–48)	OE (15) PE (32) DE (17) RE (6) SLE (7)	I (18) II (19) III (21) IV (61) Missing (2)	Proliferative (53) Secretory (81) Menstrual (14) Missing (1)	Not provided	Not provided	Finland
GSE134056	Sadia Akter	Not provided (18–49)	Not provided	Not provided	Not provided	Not provided	Not provided	USA
GSE25628 **	Stefania Crispi	31.5 (22–42)	Not provided	II (2) IV (6)	Proliferative (8)	Not provided	Not provided	Italy
GSE37837	Meraj A Khan	33.17 (24–40)	OE (18)	III (8) IV (10)	Secretory (5) Proliferative (13)	Fertile (18)	Not provided	India
GSE6364	Burney RO	36.23 (22–50)	OE, PE (7), PE (2), OE, PE, RE (5) RE, PE (7)	III* IV*	Secretory (26) Proliferative (11)	Fertile* Infertile*	Caucasian (26), Asian (4), Black (3), Asian, Indian (1) Unknown (3)	USA
GSE7305	Aniko Hever	Not provided	OE (10)	Not provided	Secretory (6) Proliferative (2) Unknown (2)	Not provided	Caucasian (10)	USA

Abbreviations in the “Type of endometriosis” columns denote ovarian endometriosis, (OE); peritoneal endometriosis, (PE); Deep infiltrating endometriosis (DIE); rectovaginal endometriosis, (RE); sacrouterine ligament endometriosis, (SLE). *No specific information regarding number of patients. **No clinical data for healthy patients (*n* = 6); clinical data available for patients with endometriosis (*n* = 8); for each affected woman, two biopsies were obtained for the eutopic and ectopic endometrium, respectively, while only one biopsy from the healthy eutopic endometrium was collected. ***For GSE153739 and GSE153740, data of 15 samples is deposited in GEO while clinical data in the corresponding article is provided for all 66 patients.

### 3.2 Transcriptomic profile of eutopic and ectopic endometrium.

**Figure 2:**
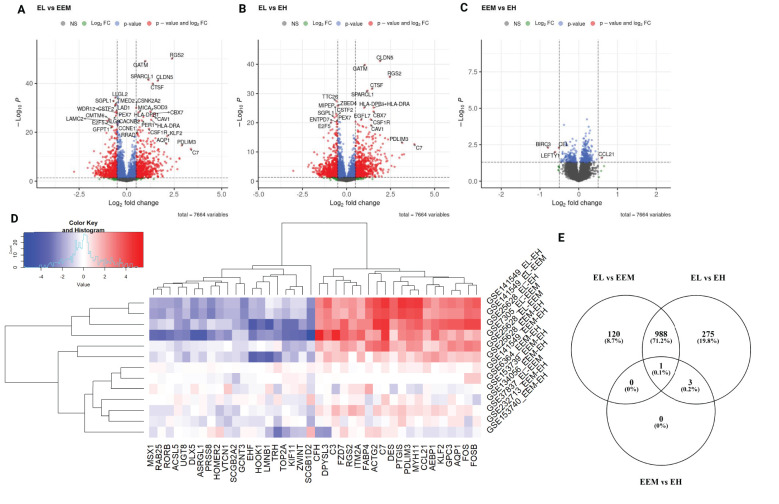
Differentially expressed genes identified by network meta-analysis. Volcano plots showing differentially expressed genes for the following comparisons **(A)** endometriotic lesions versus endometrium from women with endometriosis, **(B)** endometriotic lesions versus endometrium from women without endometriosis, **(C)** endometrium from women with and without endometriosis. For graphs A to C, red points signify genes with logFC less than −0.5 or more than 0.5 and *p*-value less than 0.05, blue points signifiy genes with logFC belonging to −0.5 to 0.5 range and p-value less than 0.05, green points signify genes with logFC less than −0.5 or more than 0.5 and p-value more than 0.05 and gray dots signify genes with with logFC belonging to −0.5 to 0.5 range and *p*-value more than 0.05. Genes with *p*-values < 1 × 10^−6^ and log2FC>|2| **(A, B)** and *p*-values < 0.05 and log2FC>|0.5| **(C)** are labeled. Heatmap with top 40 most differentially expressed genes per comparison per dataset **(D)**. Expression pattern of differentially expressed genes per comparison type **(E)**. EL, endometrial lesion; EEM, eutopic endometrium from women with endometriosis; EH, eutopic endometrium from women without endometriosis.

Differential expression analysis was performed for each of the three comparisons: EL versus EEM, EL versus EH, and EEM versus EH. Using *p* < 0.05 and |logFC| > 0.5, we identified 1,109 DEGs between EL and EEM, 1,267 DEGs between EL and EH, and four DEGs between EEM and EH (**Figure 2**). The heatmap of the top 40 upregulated and downregulated genes for all comparisons per dataset is presented in **Figure 2D**. The full list of network meta-analysis results is deposited in **Supplementary Dataset**.

The network meta-analysis revealed that the transcriptomic profile of lesions was profoundly different from that of eutopic endometrium (**Figures 2A, B**), while the eutopic endometrium from women with (EEM) and without endometriosis (EH) differed in the expression of four genes only (**Figure 2C**). *BIRC3*, *CEL*, and *LEFTY1* were significantly less expressed in the endometrium of women with endometriosis than without (logFC = −0.79, *p* = 0.0051; logFC = −0.52, *p* = 0.0051; logFC = −0.61, *p* = 0.0099, respectively). *CCL21* was significantly higher in EEM versus EH (logFC = 0.59, *p* = 0.0255) and even higher when EL with EEM was contrasted (logFC = 1.57, *p* < 0.0001, **Figure 2C**). C-C motif chemokine ligand 21 (*CCL21*) is an inflammatory mediator associated with moderate to severe endometriosis (17); however, to-date, its use as a disease biomarker has failed (18). Our results showed a directional increase of *CCL21* from the endometrium of healthy patients through that of endometriosis sufferers to lesions themselves, indicating its role in the eutopic endometrium inflammation in patients with endometriosis.

### 3.3 Pathways contributing to lesion development

**Figure 3:**
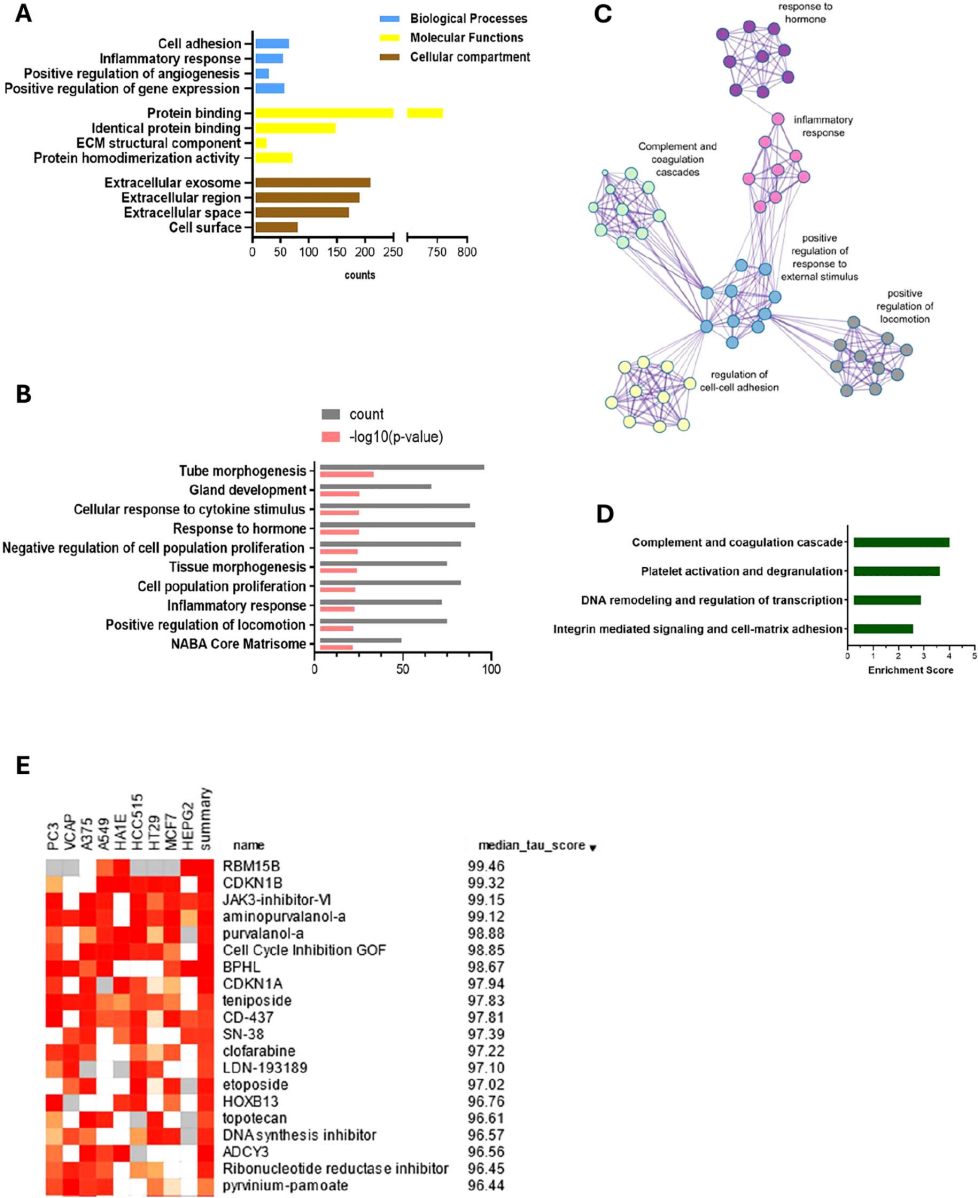
Enriched pathways analysis, functional clustering and computational pharmacogenomics of DEGs between endometriosis lesions and eutopic endometrium. Gene ontology analysis using DAVID **(A)** reveals the importance of inflammation, cell adhesion, angiogenesis and ECM remodeling. Metascape enrichment analysis **(B)** and relationship network of enriched terms visualised in Cytoscape **(C)** show key events that contribute to endometriosis development. Those include inflammatory and hormonal response and proliferation and locomotion. Functional annotation clustering reports the highest enrichment score for complement and coagulation cascade, platelet activation, DNA remodeling and integrin mediated signaling respectively **(D)**. Top 15 drug candidates identified using a drug repurposing reference database—CMap and showing median tau value above 95. JAK, CDK, and topoisomerase inhibitors are identified as potential pharmacological targets for endometriosis therapy **(E)**.

In further analysis, we selected genes that showed differential expression in both EL versus EEM and EL versus EH comparisons (the intersection of the sets, **Figure 2E**) and exhibited the same direction of expression. For *p* < 0.05 and |logFC| > 0.5, we obtained a list of 989 DEGs: 536 upregulated and 453 downregulated, on which we performed functional annotation and enrichment analyses (**Figure 3A** and detailed in **Supplementary Table S1A**). Results presented below satisfied a *p*-value below 0.0001. Those analyses revealed that most biological processes involved in the formation of endometriotic lesions were linked to cell adhesion (6.6%, *p* = 1.9 × 10^−10^), inflammatory response (5.5%, *p* = 2.8 × 10^−10^), and regulation of angiogenesis (2.9%, *p* = 7.7 × 10^−09^). The gene ontology molecular functions analysis showed that the DEGs were significantly enriched in protein binding (76.7%, *p* = 1.9 × 10^−16^), identical protein binding (15%, *p* = 2.6 × 10^−10^), and extracellular matrix structural constituent (2.5%, *p* = 4.8 × 10^−9^). In the cellular component, DEGs were mainly involved in extracellular exosome (21.2%, *p* = 2.1 × 10^−22^), extracellular region (21.2%, *p* = 2.1 × 10^−22^), and extracellular space (17.4%, *p* = 8.8 × 10^−16^).

KEGG analysis showed enrichment in complement and coagulation cascades (2.5%, *p* = 5.7 × 10^−10^), Staphylococcus aureus infection (2.3%, *p* = 1.5 × 10^−07^), and cell adhesion molecules (2.7%, *p* = 8.6 × 10^−06^). The analysis against the Reactome database revealed a key role of extracellular matrix organization (5.2%, *p* = 3.2 × 10^−10^), regulation of complement cascade (1.5%, *p* = 8.2 × 10^−07^), and complement cascade (1.6%, *p* = 2.4 × 10^−06^) (**Supplementary Table S1B**).

A further pathway enrichment analysis was performed with Metascape (14) (**Figure 3B**; **Supplementary Table S2**) and visualized in Cytoscape (15) (**Figure 3C**). Tube morphogenesis, which relates to vascular development, was the highest ranked result of enrichment analysis (**Figure 3B**). Inflammatory and hormonal response, as well as locomotion and proliferation, were among the top 10 most enriched pathways with count values above 75. Functional annotation clustering revealed that the complement cascade was the most enriched, with a score of 4.14, followed by platelet activation pathways, DNA remodeling, and regulation of transcription and cell/cell-matrix adhesion processes (enrichment scores of 3.77, 3.02, and 2.71, respectively) (**Figure 3D**).

### 3.4 Altered expression of complement and coagulation pathway genes.

**Figure 4:**
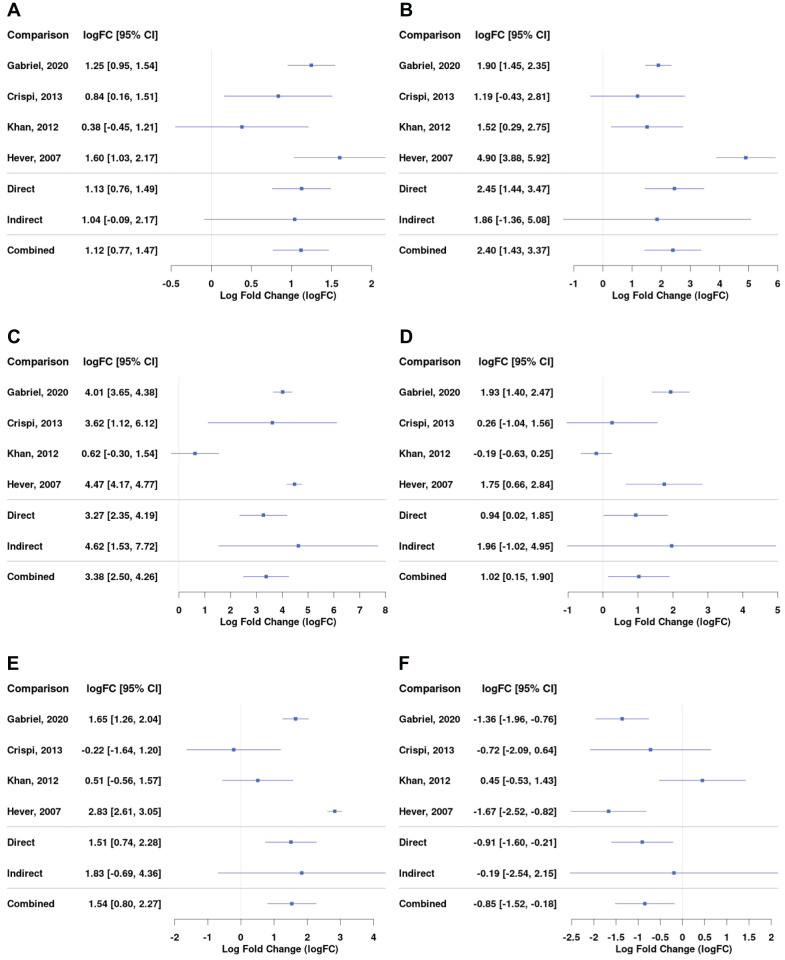
Differential gene expression across studies for selected genes from the complement and coagulation pathway for the comparison between EL versus EEM. Forest plot showing the expression of *C1QA (complement C1q A chain)* - **(A)**, *C3 (complement C3)* - **(B)**, *C7 (complement C7)* - **(C)**, *SERPINE1 (serpin family E member 1)* - **(D)**, *SERPINE2 (serpin family E member 2)* - **(E)**, and *SERPINA5 (serpin family A member 5)* - **(F)**. Direct and indirect comparisons from meta-analysis are presented in row number five and six. The indirect comparisons had a low impact on the combined comparison outcome due to the analyses being performed on datasets containing comparisons between EL and EEM (**Table 2**).

Complement and coagulation cascade was the most enriched KEGG pathway for the ectopic versus eutopic endometrium comparison (**Figure 3D**). Genes including *C7*, *C2*, *C3, A2M*, and *SERPIN* superfamily genes involved in this pathway were among the most differentially expressed in endometrial tissue (**Figure 4**; **Supplementary Dataset**).

Complement genes, including *C1QA* (logFC = 1.12; 95% CI = 0.77, 1.47), *C3* (logFC = 2.40; 95% CI = 1.43, 3.37), and *C7* (logFC = 3.36; 95% CI = 2.50, 4.26), were upregulated in endometrial lesions and showed high logFC values. *C7* was the gene that showed the highest level of upregulation in endometrial lesions among all examined genes.

Serpins regulate coagulation fibrinolysis processes (19, 20) and were implicated in the development of endometriosis (21–23). Our network meta-analysis showed that serpin genes were differentially expressed between endometrial lesions and eutopic endometrium. In comparison with the above-presented complement genes, serpin family genes were characterized by more heterogeneous expression between investigated datasets. *SERPINE1* and *SERPINE2* were upregulated (logFC = 1.02; 95% CI = 0.15, 1.90 and logFC = 1.54; 95% CI = 0.80, 2.27, respectively), while *SERPINA5* was downregulated in lesions (logFC = −0.85; 95% CI = −1.52, −0.16, **Figures 4D–F**). Detailed comparisons for each of the subgroups can be found in **Supplementary Dataset**.

### 3.5 Mast cell markers

**Figure 5:**
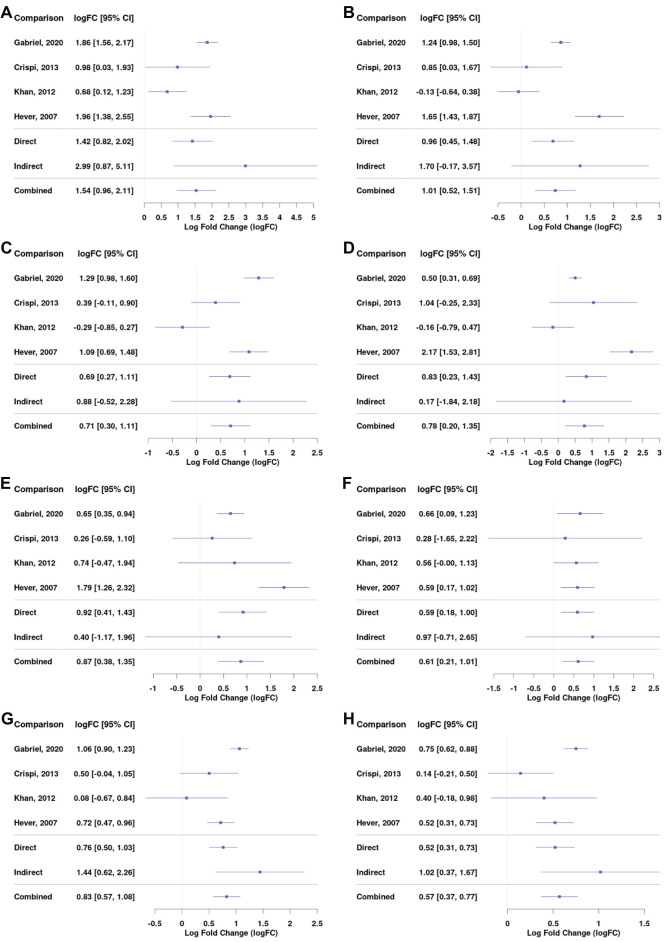
Differential gene expression across studies for selected genes, including mast cells markers, JAK/STAT pathway, and extracellular matrix markers. Forest plot showing the expression of *CPA3 (mast cell carboxypeptidase A)* – **(A)**, *KIT (tyrosine-protein kinase KIT)* – **(B)**, *MS4A6A (Membrane Spanning 4-Domains A6A)* – **(C)**, *FCGR2B (Fc Gamma Receptor IIb)* – **(D)**, *S100A10 (S100 calcium-binding protein A10)* – **(E)**, *MMP-9 (Matrix metalloproteinase-9)* – **(F)**, *STAT5A (Signal Transducer And Activator Of Transcription 5A)* – **(G)**, and *STAT5B (Signal Transducer And Activator Of Transcription 5B)* – **(H)**. Direct and indirect comparisons from meta-analysis are presented in row number five and six.

Our data showed an upregulation in the expression of mast cell markers, including *CPA3* (logFC = 1.54; 95% CI = 0.96, 2.11), *KIT* (logFC = 0.74; 95% CI = 0.30, 1.18), *MS4A6A* (logFC = 0.71; 95% CI = 0.30, 1.11), and markers of mast cell activation, *FCGR2B* (logFC = 0.78; 95% CI = 0.20, 1.35) and *S100A10* (logFC = 0.87; 95% CI = 0.38, 1.35, **Figures 5A–E**). The expression of *MS4A4A* and *MS4A2* was also higher in lesions (**Supplementary Dataset**). Higher amounts of mast cells and their increased degranulation have been reported in endometrial tissue of animal models and humans (24–26); mast cells colocalized to the vasculature of ovarian endometriomas, and they were found to promote endometrial cell migration in *in vitro* assays (27). Chromogranin and tryptase, other known markers for mast cells, were not significantly increased in lesions (**Supplementary Dataset**). Recent evidence has shown that chromogranin is not necessarily a biomarker of mast cell activation (28). Tryptase is typically assessed in patients’ serum, and its level is dependent on many factors, including genetic features and comorbidities (29), which could explain why in our comparison of tissue expression no difference was observed.

### 3.6 Repurposing JAK and CDK inhibitors for endometriosis therapy.

We used CMap drug repurposing software to find the most probable connections between therapeutic drugs and our network meta-analysis results. A median tau score value of 90 or above is considered the typical threshold for assessing meaningful drug-induced effects. We applied a median tau score cutoff at 95 and selected the top 15 hits. This analysis indicated that the candidates most likely to reverse the endometriosis mRNA profile were cyclin-dependent kinase (CDK) inhibitors, JAK, and topoisomerase inhibitors (**Figure 3E**).

The JAK/STAT3 pathway is thought to govern migratory and invasive properties of cells. Its prolonged activation in breast cancer was linked with tumor development (30) and resistance to taxane and platinum therapy (31). Our results showed an increase in the expression of *STAT5A* (logFC = 0.83; 95% CI = 0.57, 1.08) and *STAT5B* (logFC = 0.57; 95% CI = 0.37, 0.77) in lesions compared with control tissue (**Figures 5G, H**). *JAK3* significantly increased as well, but the logFC value was below 0.5 (**Supplementary Dataset**).

JAK inhibitor Ruxolitinib reduced epithelial ovarian cancer cell viability (32) and caused growth inhibition of Tam-resistant breast cancer cells *in vitro*. It was shown to lower mRNA VEGF expression and reduce the number of vessels and overall tumor weight in chorioallantoic assay (31). Ruxotinilib is currently being tested in combination therapy for endometrial cancer (33) but its use *in vitro* or in preclinical models of endometriosis has not been reported. Tofacitinib, another JAK inhibitor, showed a decrease in endometrial lesion size in mice and reduced proliferation of endometrial cancer cells *in vitro* (34).

## 4 Discussion

Understanding the main pathways involved in endometriosis development is necessary for successful biomarker discovery and improved therapy outcomes. Combining data in meta-analysis, we highlight pathogenetic mechanisms that are critical for lesion formation regardless of endometriosis subtypes and patients’ characteristics.

### 4.1 Endometrium of women with endometriosis differs from healthy controls

We detected differences in gene expression between the endometrium of healthy women and those suffering from endometriosis, thus showing that endometriosis can also affect eutopic endometrium (**Figure 2C**). *CCL21* was upregulated, while *BIRC3*, *LEFTY1*, and *CEL* were downregulated in EEM versus EH. Increased expression of *CCL21* could suggest that this gene takes part in inducing early inflammatory changes in eutopic endometrium in women with endometriosis and that it continues its role in established lesions (**Supplementary Dataset**). Baculoviral IAP repeat containing 3 (*BIRC3*) has not been studied in the context of endometriosis. However, its mutations are often present in endometrioid adenocarcinoma and endometrial cancer (35). In the latter, the lower protein levels of *Birc3* correlate with worse patient survival (36). One could speculate that the decreased *Birc3* expression in EEM may contribute to the transformation of the endometrium into lesions. Endometrial bleeding associated factor (*EBAF*/*LEFTY1*) partakes in the regulation of cyclical exfoliation of endometrium and in decidualization (37). Healthy endometrium does not express *LEFTY1* during the implantation window, while the endometrium of women suffering from endometriosis as well as infertility showed its expression (38). Our results agree with that finding and suggest that the higher *LEFTY1* expression in the EEM group could contribute to endometriosis-related infertility. The *CEL* gene encodes carboxyl ester lipase, which partakes in cholesterol and lipid-soluble vitamin ester hydrolysis. Its role so far is implicated in diabetes and hereditary pancreatitis (39–41) and progression of atherosclerosis. The *CEL* gene has not yet been studied in the context of endometriosis.

### 4.2 The complement and coagulation cascade in lesion formation.

We further focused on delineating the expression profile that can differentiate ectopic endometrium from eutopic endometrium from women with and without endometriosis (**Figure 2**). Gene ontology analyses highlighted crucial events accompanying lesion formation. Those were immune system activation, angiogenesis, regulation of transcription, response to hormones and cytokines, cell adhesion, and ECM-cell surface interactions (**Figure 3**). The importance of immune system deregulation in endometriosis has been reported previously; various inflammatory phenotypes have been associated with increased risk of endometriosis (1–3). Our result showed that the complement system and platelet coagulation are the two most enriched pathways in endometriosis (**Figure 3D**). Both processes are essential in the natural endometrium growth and shedding cycle. The fact that both pathways are the most enriched agrees with the current theory that women prone to endometriosis are likely to have a different, dysregulated peritoneal microenvironment. The complement system is a mediator of tissue growth and regeneration (42) and its activation has for a long time been implicated in the development of autoimmune disease (43, 44) and in promoting tumor growth (45). Its dysregulation could therefore provide means for immunosurveillance escape and facilitate the implantation of lesions. Its importance in the development of endometriosis has been suspected since the 80’s (46) and confirmed more recently (47, 48). Higher amounts of C1, C3, and C5 have been detected in serum (49) and peritoneal fluid of women with endometriosis (50, 51). Various complement proteins were shown to be present in epithelial cells of endometrial lesions and ovarian cancer tumors. Its local synthesis and deposition have been correlated with the progression of various cancer types (52).

Our data revealed an increased mRNA expression of *C1q*, *C2*, and *C6*, but especially *C3* and *C7*, in the lesions (**Figures 4A–C**; **Supplementary Dataset**). *C7*, a complement cascade member responsible for initiation of the membrane attack complex, was the most overexpressed gene with the highest fold change in our comparison between diseased and control tissue, suggesting its significant role in lesion formation (**Figures 2A–C**). C7 was found to contribute to inflammation and tissue damage in endometriosis (53); it has previously been shown to be overexpressed in ovarian cancer (2) and stromal cells of endometriomas (3).

C3, a major effector at which all complement pathways converge, was one of the most differentially expressed genes in endometriosis (**Figure 4B**; **Supplementary Figure S1**). C3 dysregulation is involved in most, if not all, inflammatory diseases; it has been found upregulated in cancer, cardiac and neurological diseases, asthma, and obesity (54–58). Patients with inflammatory bowel disease had a higher expression of C3 in their intestinal tissue, and this is thought to contribute to chronic inflammation and tissue injury (59). Local C3 deposition has been suggested as a prognostic factor for gastric cancer (60). A similar situation could occur in endometriosis; increased C3 expression could contribute to inflammation-driven peritoneal tissue injury, which in turn would facilitate lesion implantation. Glandular epithelial cells found in endometrial lesions were shown to produce C3 locally (61). The activity of both complement cascade members C3 and C4 was higher in the serum of women with endometriosis than in those without (62). Increased amounts of C1, C3, and C5 were detected in serum (49) and peritoneal fluid of women with endometriosis (50, 51). Similarly, in lesion-bearing mice, C3 was increased in their peritoneal fluid. Animals with *C3* knockdown formed smaller endometrial cysts and, on average, fewer of them (61).

C3 seems pivotal to endometriosis pathology, and given its strong upregulation and presence both in tissue (**Figure 4B**) and well in peritoneal fluid of endometriosis sufferers, it poses an interesting target for early diagnosis and therapy. It has already been proposed as an endometriosis serum biomarker (63). However, due to the observed discrepancies between tissue and plasma levels of C3, further investigation is necessary. In gastric cancer, C3 tissue deposition showed a negative correlation with plasma levels, highlighting the need for additional research to determine whether C3 is a suitable biomarker for endometriosis. As far as treatment is concerned, C3 inhibitors have entered clinical trials in anti-ovarian cancer therapy (64) and treatment against inflammatory bowel disease (59). Our results indicate that the biomarker and therapeutic potential of C3 should be studied in endometriosis in more depth.

Our results revealed strong enrichment in the coagulation cascade and showed a dysregulation of *SERPIN* superfamily genes in endometrial lesions (**Figures 3D**, **4D–F**; **Supplementary Figure S1**), suggesting an imbalance in the coagulation-fibrinolysis processes (19, 20).

*SERPINE1* and *SERPINE2* were increased in endometrial lesions (**Figures 4D, E**). *SERPINE1-*encoded PAI-1 inhibits fibrinolysis and contributes to thrombosis/fibrosis. *PAI-1* was shown to stimulate angiogenesis and facilitate tumor growth and metastasis in primary neuroblastoma tumors (65) and in ovarian cancer, its increased expression was correlated with tumor cell proliferation and overall poor prognosis. The inhibition of *PAI-1* suppressed ovarian cancer cell growth (66). Its role in endometriosis has also been suggested; by inhibiting peritoneal fibrinolysis, *PAI-1* is thought to contribute to the formation of endometriotic adhesions. *PAI-1* was found to be increased in deep infiltrating endometriosis as compared to other subtypes and eutopic endometrium (67). Moreover, endometriosis patients treated with dopamine receptor 2 agonist—quinagolide, showed decreased expression of tissue *PAI-1* accompanied by a decrease in lesion size or its complete disappearance (68). Our results further support the importance of *PAI-1* in lesion maintenance.

*SERPINE2* was implicated in modulating DNA damage response (69) and favoring cancer cell invasion (70). Its pro-metastatic activity has been linked to extracellular matrix remodeling and an increase in matrix metalloproteinase 9 (MMP-9) expression (71, 72). High *SERPINE2* levels in the endometrium during the secretory phase suggest its involvement in tissue remodeling during implantation (73). In a mouse model of endometriosis, *SERPINE2* showed upregulated expression (74); its role in human endometriosis remains unstudied.

Reduction in *SERPINA5* expression was linked with an aggressive tumor phenotype and poor prognosis in endometrial and ovarian serous carcinomas (75, 76). The delivery of *SERPINA5* through exogenous exosomes decreased the migratory potential of endometrial cancer cells (76). Similarly, overexpression of *SERPINA5* resulted in decreased invasion and angiogenesis in breast cancer (77). Decreased *SERPINA5* expression was correlated with downstream activation of MMP9 in ovarian serous carcinomas (78). Our meta-analysis revealed lower *SERPINA5* and higher *MMP9* expression in endometrial lesions (**Figure 4F**). Moreover, ECM interactions were indicated in enrichment analysis (**Figures 3A, B, D**). Taken together, our results suggest that the imbalance in the coagulation pathway may be affecting extracellular matrix remodeling and contributing to the metastatic-like potential of endometriotic cells, thereby promoting lesion formation.

### 4.3 JAK/STAT3 pathway inhibition

Our search for associations between the endometriosis gene signature and CMap reference perturbagens highlighted the role of inhibitors of JAK, CDK, and topoisomerase as possible therapy candidates (**Figure 3E**). JAK/STAT3 pathway dysregulation correlated with an increased proliferation and angiogenesis in cancer (79) and with various immunodeficiency syndromes (80, 81).

Our meta-analysis revealed an increased expression of both *STAT5A* and *STAT5B* in lesions compared with control tissue (**Figures 5G, H**). Others have shown that phosphorylation of STAT3 was upregulated in endometriosis lesions (82) and activated STAT3 increased proliferation of endometrial stromal cells (83).

Interestingly, increased C3 expression was shown to trigger the JAK/STAT3 pathway in gastric cancer, which led to a subsequent increase in cell proliferation. C3 inhibition with CR1 decreased that activation (60). Our results present a similar picture; complement C3 as well as the JAK/STAT3 pathway seems to play a role in the development of endometriosis. This association needs further investigation.

### 4.4 Proposed pathways crosstalk in endometriosis

**Figure 6:**
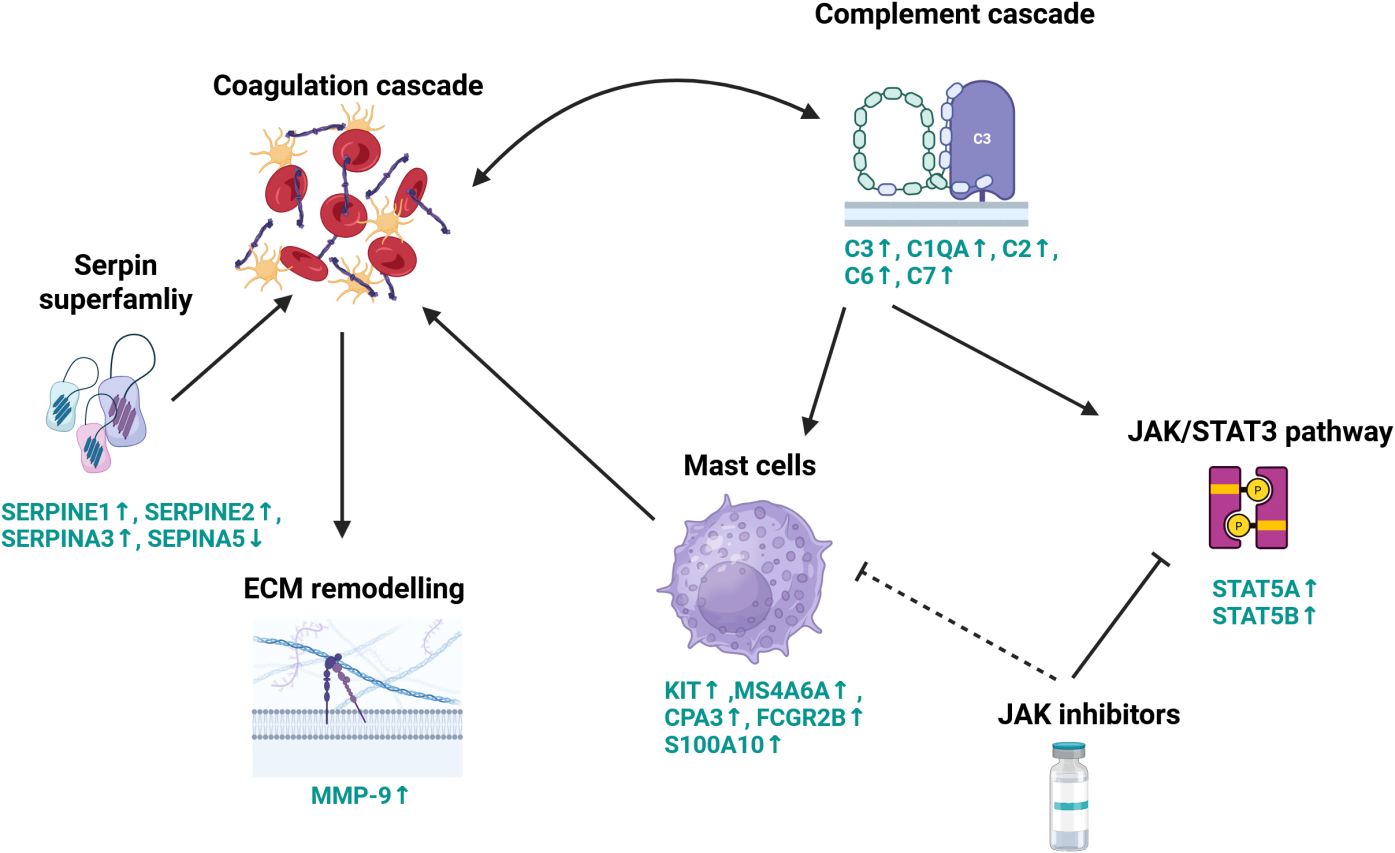
Schematic representation of proposed key molecular processes contributing to lesion development. This diagram illustrates the complex interplay between the complement system, coagulation cascade, extracellular matrix remodeling, and immune signaling pathways in the context of lesion formation. Coagulation factors cleave and activate complement components C3 and C5, initiating pro-inflammatory responses. Complement factor C3 also functions to stabilize clots by protecting them from fibrinolysis. The coagulation cascade is tightly regulated by members of the serpin superfamily, which modulate extracellular matrix remodeling through the induction of matrix metalloproteinase-9 (MMP-9) expression. Complement activation further engages the JAK/STAT signaling pathway, particularly STAT5, promoting mast cell activation. Activated mast cells, in turn, influence coagulation dynamics. Pharmacologic inhibition of the JAK/STAT pathway using JAK inhibitors can suppress STAT3 signaling and reduce mast cell degranulation, thereby modulating both inflammatory and thrombotic processes. These mechanisms highlight the therapeutic potential of JAK inhibitors in the context of endometriosis. Genes names in green signify differentially expressed genes, arrows show the directional change in gene expression in endometriosis lesions compared with control endometrium. Created in BioRender.com.

It has been proposed that both the complement system and coagulation pathways are tightly linked; coagulation factors have been reported to cleave and activate complement members C3 and C5 (84). On the other hand, C3 was shown to protect clots from fibrinolysis (85). Increased amounts of C3 protein were shown to provoke mast cell activation, and various mast cell mediators were implicated in the regulation of coagulation and fibrinolysis in anaphylaxis (86). Our meta-analysis revealed that endometrial lesions had a higher expression of mast cell markers, including *KIT*, *CPA3*, *MS4A6A*, *FCGR2B*, and *S100A10* (**Figures 5A–E**). An increased mast cell burden was detected previously in animal and human endometrial tissue (27). Moreover, our results showed that endometrial lesions had a higher level of *STAT5A* and *STAT5B* (**Figures 5G, H**), members of the JAK/STAT3 pathway, which regulate mast cells (87). Targeting mast cells with JAK inhibitors for alleviation of symptoms of endometriosis was proposed almost two decades ago (88) but not much research has been carried out on the topic since. Our current results fill this gap and suggest the use of JAK inhibitors as immunomodulators in endometriosis. Interestingly, a cooperation between mast cells, complement, and coagulation pathways has been reported in an inflammatory disease—chronic spontaneous urticaria (89). Our analysis indicates that there exists an interplay between the complement and coagulation pathways, mast cell activation, ECM remodeling, and the JAK/STAT3 pathway (summarized in **Figure 6**). To the best of the author’s knowledge, this relationship has not yet been studied in endometriosis, and our results warrant a further in-depth look into those processes.

### 4.5 Strengths and limitations

Our network meta-analysis enabled us to arrive at a consensus endometriosis signature. The use of publicly deposited endometriosis transcriptomic data collected on three different continents, spanning various age groups, and ethnicities, as well as various types and stages of endometriosis, enabled a comprehensive, unbiased, and multi-demographic comparison of endometriotic and control tissue.

The following limitations should be considered when interpreting our results. Our meta-analysis included only nine datasets because most of the studies lacked a control group, included therapeutic intervention, or performed RNA isolation on processed tissue. Secondly, only published studies where the absence of endometriosis was excluded by laparoscopy were included in this meta-analysis. Therefore, publication bias may have occurred, although none was indicated by the funnel plot.

### 4.6 Conclusions and clinical implications

We highlight the role of complement and coagulation cascade in endometriosis and propose an interplay between both those processes and mast cells, ECM interaction, and the JAK/STAT3 pathway that needs further investigation. We underscore the significance of C3 and call for further research into its diagnostic and therapeutic potential. Furthermore, we propose JAK inhibitors discovered in drug repurposing analysis and validated *in vitro* as potential therapy candidates.

Our results show differences in expression in eutopic endometrium from patients with and without endometriosis. Those should be further explored to understand if they contribute to endometrial seeding. Detected gene differences may be potential biomarkers that could be used in the less invasive endometriosis biopsy and should be further studied.

## Data Availability

The original contributions presented in the study are included in the article/**Supplementary Material**. Further inquiries can be directed to the corresponding author.
