# Supplementary material for: Complement and coagulation cascade cross-talk in endometriosis and the potential of Janus Kinase inhibitors—a network meta-analysis
Source: Front Immunol. 2025 Jul 8;16:1619434. doi: 10.3389/fimmu.2025.1619434 (PMC12279843; doi:10.3389/fimmu.2025.1619434)
Supplement: Supplementary file 2 [file Table1.docx]

**SUPPLEMENARY MATERIAL**: **Complement and coagulation cascade cross-talk in endometriosis and the potential of JAK inhibitors – a network meta-analysis.**

| **A** | | | | |
| --- | --- | --- | --- | --- |
| **Gene ontology term** | **Gene count** | **%** | **P-Value** | **FDR** |
| ***Biological Processes*** |  | | | |
| cell adhesion | 65 | 6,6 | 1,9E-10 | 6,4E-07 |
| inflammatory response | 54 | 5,5 | 2,8E-10 | 6,4E-07 |
| positive regulation of angiogenesis | 29 | 2,9 | 7,7E-09 | 1,2E-05 |
| positive regulation of gene expression | 57 | 5,8 | 1,4E-08 | 1,6E-05 |
| kidney development | 23 | 2,3 | 7,9E-08 | 7,2E-05 |
| ***Molecular Functions*** |  | | | |
| protein binding | 759 | 76,7 | 1,9E-16 | 2,2E-13 |
| identical protein binding | 148 | 15 | 2,6E-10 | 1,5E-07 |
| extracellular matrix structural constituent | 25 | 2,5 | 4,8E-09 | 1,9E-06 |
| protein homodimerization activity | 72 | 7,3 | 2,3E-07 | 6,6E-05 |
| heparin binding | 27 | 2,7 | 1,3E-06 | 3,1E-04 |
| ***GO cell component*** |  | | | |
| extracellular exosome | 210 | 21,2 | 2,1E-22 | 1,4E-19 |
| extracellular region | 190 | 19,2 | 5,3E-17 | 1,7E-14 |
| extracellular space | 172 | 17,4 | 8,8E-16 | 1,9E-13 |
| cell surface | 80 | 8,1 | 1,3E-14 | 2,1E-12 |
| plasma membrane | 352 | 35,6 | 1,6E-11 | 2,1E-09 |
| **B** | | | | |
| **Gene ontology term** | **Gene count** | **%** | **P-Value** | **FDR** |
| ***Pathways (KEGG pathway)*** |  | | | |
| Complement and coagulation cascades | 25 | 2,5 | 5,7E-10 | 1,9E-07 |
| Staphylococcus aureus infection | 23 | 2,3 | 1,5E-07 | 2,5E-05 |
| Cell adhesion molecules | 27 | 2,7 | 8,6E-06 | 9,5E-04 |
| Human T-cell leukemia virus 1 infection | 33 | 3,3 | 1,6E-05 | 1,1E-03 |
| Hematopoietic cell lineage | 20 | 2 | 1,7E-05 | 1,1E-03 |
| ***Pathways (REACTOME pathway)*** |  | | | |
| Extracellular matrix organization | 51 | 5,2 | 3,2E-10 | 4,9E-07 |
| Regulation of Complement cascade | 15 | 1,5 | 8,2E-07 | 6,4E-04 |
| Complement cascade | 16 | 1,6 | 2,4E-06 | 1,3E-03 |
| Molecules associated with elastic fibres | 12 | 1,2 | 1,3E-05 | 4,3E-03 |
| Elastic fibre formation | 13 | 1,3 | 1,4E-05 | 4,3E-03 |

**Supplementary Table S1**. Top 5 enriched GO terms per Biological Processes, Molecular functions and Cell Component category (A) and KEGG and REACTOME pathways (B).

| GO | Description | Count | % | Log10(P) | Log10(q) |
| --- | --- | --- | --- | --- | --- |
| GO:0035239 | tube morphogenesis | 96 | 9.71 | -33.30 | -28.95 |
| GO:0048732 | gland development | 66 | 6.67 | -25.31 | -21.61 |
| GO:0071345 | cellular response to cytokine stimulus | 88 | 8.90 | -25.21 | -21.61 |
| GO:0009725 | response to hormone | 91 | 9.20 | -25.17 | -21.61 |
| GO:0008285 | negative regulation of cell population proliferation | 90 | 9.10 | -24.32 | -20.82 |
| GO:0048729 | tissue morphogenesis | 75 | 7.58 | -24.05 | -20.60 |
| GO:0008283 | cell population proliferation | 83 | 8.39 | -23.21 | -19.81 |
| GO:0006954 | inflammatory response | 72 | 7.28 | -22.44 | -19.14 |
| GO:0040017 | positive regulation of locomotion | 75 | 7.58 | -21.84 | -18.57 |
| M5884 | NABA CORE MATRISOME | 49 | 4.95 | -21.60 | -18.40 |
| GO:0022407 | regulation of cell-cell adhesion | 66 | 6.67 | -21.55 | -18.38 |
| R-HSA-1474244 | Extracellular matrix organization | 51 | 5.16 | -21.51 | -18.37 |
| GO:0030855 | epithelial cell differentiation | 75 | 7.58 | -21.42 | -18.31 |
| GO:0032103 | positive regulation of response to external stimulus | 70 | 7.08 | -21.21 | -18.12 |
| GO:0061061 | muscle structure development | 66 | 6.67 | -20.42 | -17.42 |

**Supplementary Table S2**. Top 15 clusters enriched in endometrial lesions with their representative enriched terms. Count refers to the number of genes from the user-provided lists that are associated with the specified ontology term. % represents the proportion of all user-supplied genes that are included in that ontology term (only genes with at least one ontology annotation are considered in the calculation). Log10(P) is the p-value expressed in base-10 logarithmic form. Log10(q) is the p-value adjusted for multiple testing, also in base-10 logarithmic form.


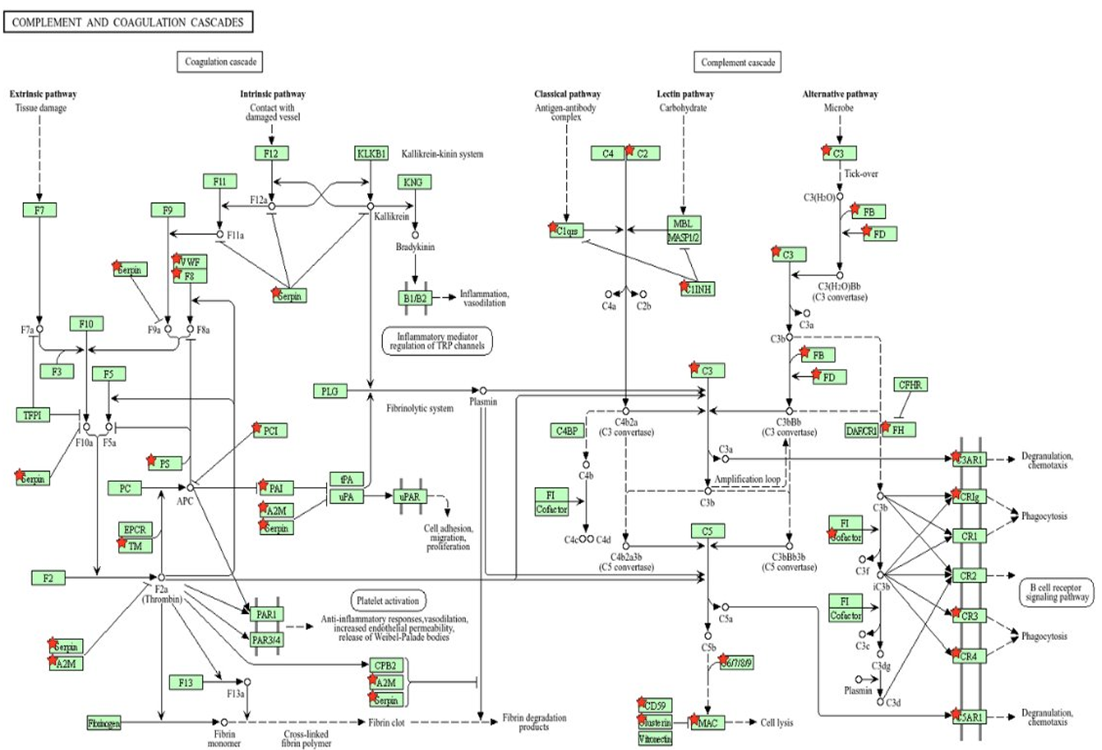


**Supplementary Figure S1**. Enrichment in the complement and coagulation pathway based on KEGG pathway analysis performed in DAVID. Pathway analysis against KEGG database showed that complement and coagulation cascades are the most enriched molecular events, red stars denote DEGs. Genes responsible for this enrichment include C2, C3, SERPINS and A2M.
